# Intermittent Auscultation in Labor: Could It Be Missing Many Pathological (Late) Fetal Heart Rate Decelerations? Analytical Review and Rationale for Improvement Supported by Clinical Cases

**DOI:** 10.14740/jocmr2298w

**Published:** 2015-10-23

**Authors:** Shashikant L. Sholapurkar

**Affiliations:** Department of Obstetrics and Gynaecology, Royal United Hospital NHS Foundation Trust, Bath, UK. Email: s.sholapurkar@nhs.net

**Keywords:** Intermittent auscultation, Electronic fetal monitoring, Fetal heart rate decelerations, Intrapartum fetal monitoring, Intrapartum fetal surveillance

## Abstract

Intermittent auscultation (IA) of fetal heart rate (FHR) is recommended/preferred in low risk labors. Its usage even in developed countries is poised to increase because of perceived benefit of reduction in operative intervention and some disillusionment with the cardiotocography (CTG). Many national guidelines have stipulated regimes (frequency/timing) of IA based on level IV evidence. These tend to get faithfully and exactingly followed. It was observed that deliveries of many unexpectedly asphyxiated infants occurred despite rigorously performed and documented IA compliant with the guidelines. This triggered a reappraisal of the robustness of IA leading to this focused review supplemented by two anonymized cases. It concludes that the current methodology of IA may be flawed in that it poses a risk of missing many or most late (pathological) FHR decelerations, one of the foremost goals of IA. This is because many late decelerations reach their nadir before the end of the contraction. Thus the currently recommended auscultation of FHR for 60 seconds after the contraction by all national guidelines seemed to encompass their “recovery” phase and appeared to be misinterpreted as normal FHR or even as a reassuring accelerative pattern in the clinical practice. A recent recommendation of recording of the FHR as a single figure (rather than a range) does not remedy this anomaly and seems even less informative. It would be better to auscultate FHR before and after the contractions (or contraction to contraction) and take the FHR just before the contraction as the baseline FHR and interpret the FHR after contraction in the context of this baseline. This relatively simple improvement would detect most late FHR decelerations thus ameliorating the risk and significantly enhancing the patient safety.

## Introduction

Intermittent auscultation (IA) of fetal heart rate (FHR) is widely practiced in low risk labors in many countries. The National Institute for Health and Clinical Excellence, UK (NICE, 2014) strongly recommends IA for up to 45% of all labors because it has been shown to reduce the operative intervention and is generally regarded to be safe for the babies [1]. On the other hand, cardiotocography (CTG) is the norm in the USA [2] on the grounds of its perceived superiority and higher reliability based on day to day clinical experience/observation (but not necessarily good quality evidence) as well as for medico-legal defence. However, the CTG has been the main driver of medico-legal claims for hypoxic neurological injury over the last 50 years rather than protection against it [3]. Moreover, the three tier systems of FHR pattern interpretations have been found wanting in guiding the clinical decisions to reduce birth asphyxia [4-6]. A systematic review of randomized controlled trials (RCTs) reported that IA is as good as continuous CTG in the low risk labors [7]. The national guidelines of Canada, Australia-New Zealand and UK recommend auscultation for 60 s after a contraction every 15 - 30 min (based on expert consensus only), intended to detect late pathological FHR decelerations as confirmed by NICE [1, 8-10]. The birth attendants are expected to rigorously adhere to these guidelines and indeed do so. However, most obstetric units including ours continue to encounter unexpected deliveries of asphyxiated infants despite rigorously performed and documented IA compliant with the guidelines. This is generally regarded as unexplained and possibly even unavoidable. However, this did trigger in depth rethinking about IA. On several occasions birth attendants from different hospitals have come across patients who have had normal IA, but were found to have pathological FHR pattern (especially late decelerations) as soon as they were placed on CTG. Poor neonatal outcome resulted in some cases despite prompt delivery, leading to a conjecture that IA may have missed the abnormal FHR pattern for quite some time in those labors. Out of many such occurrences from different hospitals, two anonymized but real clinical cases are presented here as illustrations. These raise a significant possibility (even if not a proof beyond doubt, which may be unattainable) that there are correctible methodological flaws in the current IA which could have led to severe birth asphyxia. A peculiar attribute of IA is that its “correctness” or “reliability of its performance” cannot be objectively scrutinized retrospectively (apart from the documented “normal” FHR). Any RCTs or good quality cohort studies on IA are lacking in the last 15 years in the literature because these are particularly difficult to conduct and resource intensive. However, there is scope to improve safety and reliability of IA by making relatively simple changes based on the analytical review and debate presented here. This debate is all the more important because most obstetricians from developed countries almost completely lack practical experience in performing IA which is mostly a domain of midwives especially in the UK. As a result, the obstetricians are likely to devote little attention or interest to the intricacies of IA, despite its increasing application. This review has a limited scope with focus on the different regimes of IA recommended by most national guidelines (not any particular one) for low risk labors. Other aspects like for example the high/low risk categorization of the labor are outside the scope of this review.

## The Basis for Regimes of IA

IA of FHR in low risk labors has been practiced for several decades. Before the turn of the century, different hospitals had their own slightly varied protocols about the methodology of IA. Fortunately, the incidence of moderate/severe birth asphyxia in the absence of risk factors or an acute intrapartum adverse event is very low although not insignificant. At one extreme, a few regimes of IA can be quite loose or relaxed accepting this risk. For example, in Netherlands where all home births receive IA only, no structured guidelines are followed and as a convention FHR is auscultated every 2 h or so in the active first stage (personal correspondence) [11]. However, a more proactive approach is preferred by most. Thus, with the aim to achieve standardization and improvement of care, specific guidelines for IA have been issued, e.g. by the Royal College of Obstetricians and Gynaecologists (RCOG, 2001; NICE, 2014), American Congress of Obstetricians and Gynecologists (ACOG, 2009), the Royal Australian and New Zealand College of Obstetricians and Gynaecologists (RANZCOG, 2014) and the Society of Obstetricians and Gynaecologists of Canada (SOGC, 2007) [1, 2, 8-10]. These guideline development groups were unable to find any studies correlating different protocols for timing and frequency of IA to the neonatal and maternal outcomes [1, 8, 9]. Hence, the recommendation of the frequency and timing of IA is mostly based on collective reasoning or level IV evidence. To take an example, the RCOG (2001) made following recommendations which have been endorsed by NICE [1, 8].

“In the active stages of labor, IA should occur for 60 s after a contraction, every 15 min in the first stage, and every 5 min in the second stage. Continuous CTG should be offered, if there is evidence on auscultation of a baseline less than 110 bpm or greater than 160 bpm, auscultation of any decelerations, and if any intrapartum risk factors develop.”

The other national guidelines have made very similar recommendations. The most important consideration behind the timing of IA is to detect late (pathological) FHR decelerations [1]. In 2014, NICE made a new recommendation that the FHR should be recorded as a single rate (rather than a range) auscultated over 1 min after contraction [1]. There seems a lack of clarity as to which figure to choose when a hand held Doppler device is used to auscultate FHR, as is the common practice. If the findings of IA are abnormal, then all national guidelines recommend CTG. NICE recommends CTG for 20 min and if no further abnormalities are observed, then CTG should be discontinued and IA recommenced [1] which seems a very rational approach.

## Supporting Evidence From Clinical Practice

### Case 1

**Figure 1:**
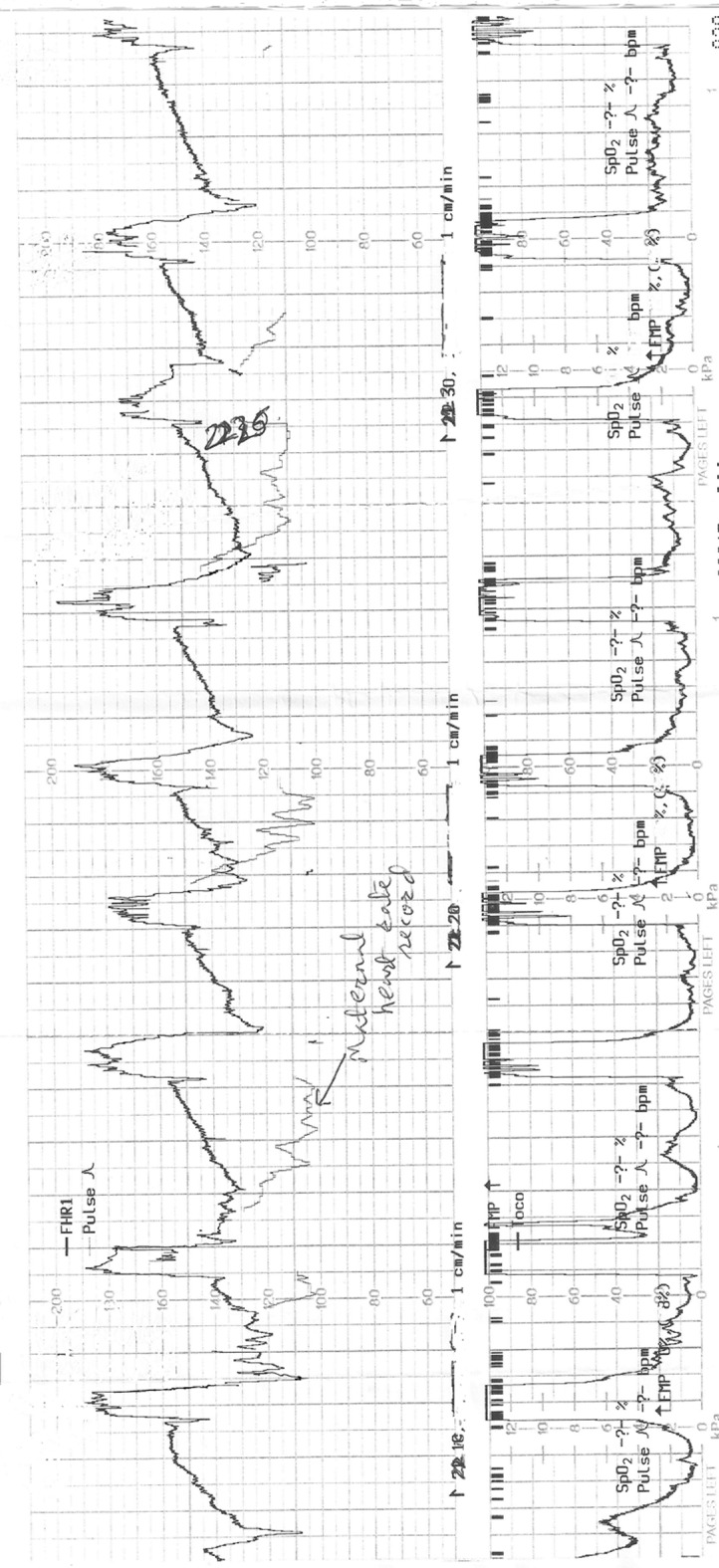
The cardiotocograph (CTG) in case 1 showing recurrent pathological FHR decelerations of late onset and gradual late recovery to baseline, which were missed on intermittent auscultation (IA). CTG paper recording speed 1 cm/min.

A 34-year-old nulliparous lady with previous one miscarriage presented in early labor at term with cephalic presentation. In view of low risk status, IA of FHR was performed and documented every 15 min during the first stage and every 5 min in the second stage. The documentation in the first and second stage read, “FHR for 60+ s following contractions as 120 - 132, 125 - 144, 116 -136; no decelerations”. At times it was documented, “FHR accelerates from 120 to 140 (118 - 138, 122 - 138 accelerative) over 60 s following contraction”. Since the case predated the current NICE (2014) guidelines, the FHR was documented as a range or trend over the 60 s [1]. Based on current guidelines (NICE, 2014) [1], the FHR could have been documented as a single figure of about 130/min, well within normal range but probably less informative than a trend. There was no meconium or any other high risk factor noted. After pushing for 2 h in the second stage, a CTG was commenced in case operative delivery was required (Fig. 1). The CTG assessment 30 min later documented reduced baseline variability and “atypical variable” decelerations (fairly common and unhelpful categorization between 2007 and 2015) from the beginning of the CTG. However, these FHR decelerations were described as “late” in retrospect during the “case review” in the multi-professional perinatal morbidity meeting held subsequently. An obstetrician was called who performed a straightforward ventouse delivery in view of a “suspicious” CTG and prolonged second stage. A normal size baby was delivered in an unexpected poor condition (low Apgar score, umbilical arterial pH below 6.95 and base excess -14; venous pH 7.00 and base excess -14). The baby developed grade 3 hypoxic-ischemic encephalopathy (HIE) with seizures and underwent therapeutic hypothermia (head cooling) and was discharged several days later with very guarded long-term prognosis.

### Case 2

A 32-year-old woman presented at term pregnancy with cephalic presentation and infrequent mild labor pains (low risk) in a community midwifery unit. On admission, FHR was auscultated with Doppler over several minutes and documented to be 130 - 140 bpm. An examination showed her to be in the latent phase of labor. An hour later, patient complained of rectal pressure and the cervix was found to be fully dilated. IA of FHR was commenced every 5 min for 60 s following contraction. Predating to the current NICE (2014) guidelines, the FHR on IA was documented as a range rather than a single figure [1]. Several documentations of FHR read, “110 - 128, 111 - 123, 115 - 125, 110 - 130, 120 - 126 and so on, accelerative, no decelerations heard”. There was no meconium or any other high risk factor noted. After 2 h of pushing, a normal size baby was delivered with unexpected low Apgar scores. The baby was resuscitated but remained “floppy” and was transferred to the hospital neonatal intensive care unit. Cord blood gas testing was not available in the community unit. However, the baby developed grade 3 HIE indicating strong possibility of intrapartum asphyxia. In view of irritability, hypertonia (HIE) and abnormal cerebral function monitoring (CFM) recording, the baby underwent therapeutic head cooling and re-warming over several days. Baby was discharged with guarded long-term prognosis.

## Lessons From the Clinical Practice

It would be worth examining the inferences from the two anonymized clinical cases presented above. Although the IA was performed in these low risk labors fairly rigorously complying with the national guidelines, the babies were born unexpectedly with birth asphyxia leading to significant neonatal morbidity. In the first case, the CTG (Fig. 1) when commenced showed pathological decelerations (late) right from the outset, even though the IA had not identified any decelerations. This CTG tracing (Fig. 1) is of a limited quality because the mother was pushing and there is superimposed record of maternal heart rate. However, it clearly shows recurrent FHR decelerations of 20 - 40 bpm depth, with onset consistently late in relation to the onset of contractions. It is important to note that the troughs of the most decelerations are already reached before the end of the contractions. If one concentrates on the “60 s post-contraction interval” (where IA is recommended to be sufficient), what can be seen is the partial recovery of FHR by 15 - 20 bpm (Fig. 1). But the recovery is not complete for further 1 - 2 min almost until the beginning of the next contraction. It is remarkable to note that this is precisely what the midwives repeatedly recorded during IA, i.e. normal FHR of about 120 - 140 bpm which was often perceived and documented as “normal” or “accelerative”! Hence it may be possible to conclude that IA for 60 s after contractions may not have diagnosed these grossly pathological (late) decelerations which are one of the foremost goals of IA. If the FHR returns to an abnormally high baseline (tachycardia) later than the 60 s after the end of contractions but before the next contraction, then this baseline tachycardia would also be missed by the current practice of IA. However, it would be possible to detect this if the auscultation is performed just before the contraction. A critical analysis of the CTG in case 1 (Fig. 1) suggests that the late decelerations may have been suspected on IA if auscultation was performed before and after contractions, or from contraction to contraction, and the FHR just before the contractions was taken as the “baseline FHR”, and the FHR after the contractions was interpreted in the context of this baseline. Alternatively if the auscultation is performed during and after contractions, then also these late decelerations would have been detected. However, in that case to avoid unnecessary intervention, the FHR decelerations limited to and coinciding with contractions should be disregarded as previously because they are benign [5, 6, 12].

The second case does not include a CTG (was not performed) which highlights the common impediment in scrutinizing IA. However, the rigorously performed IA documents very similar record of FHR as in the first case (e.g. 110 - 130 bpm) over 60 s after the contractions also described as “accelerative” on quite a few occasions. Moreover, there was one record of FHR 130 - 140 bpm only 1 h before full dilatation and 3 h before delivery. If this was the baseline FHR, then it seems a distinct possibility that the midwife could have been listening on recovering late decelerations without realization. However, there is no recommendation in the current training or IA guidelines for establishing the baseline FHR [1, 8, 10]. Even if the baseline FHR is established by auscultation at the time of admission [9], there is no mention or advice in the guidelines about relating the subsequent FHR on IA to this baseline [9]. Moreover, the baseline FHR is likely to change/rise over the course of labor and hence repeated confirmation/assessment of baseline FHR would be clearly desirable [12]. The cases presented above do not provide a proof beyond doubt but do seem logically to point to serious (correctible) deficiencies in the IA regimes impairing patient safety.

## Perception of the Obstetricians and Midwives

The obstetric experts from developed countries generally have hardly any practical experience of IA while midwifery staff are under pressure to follow guidelines rigidly despite the onerous demands of auscultation every 15 min in labor. On discussion with midwives and obstetricians, there seems an expectation of either a clear deceleration being heard after the end of palpable contractions (i.e. a drop in FHR followed by rise) or an abnormal FHR (e.g. below 110) for IA to be considered abnormal, both relatively rare. This expectation is of course bolstered by the current guidelines [1, 8-10]. On the other hand, a more likely pathological scenario in the 60 s after contraction is likely to be recovery of a late deceleration with nadir already reached in the later part of the contraction. This more common pathological FHR pattern would be missed by the current recommended practice of listening for 60 s after contractions only. Even a more serious combination of baseline tachycardia and late decelerations may be missed as alluded previously in the first clinical case. In 2014, the NICE made a new recommendation that the FHR should be recorded as a single figure (rather than a range) over 60 s after a contraction [1]. The senior midwives interpret this as counting FHR over 60 s by a (Pinard or standard) stethoscope which would represent the “average FHR” during the 60 s. The rationale behind this is quite inexplicable, because not only a recovering late deceleration may be missed but even if FHR drops from a baseline of 150/min to 120/min and then rises again to 150/min during the 60 s after contraction (a pronounced late decelerations); the FHR counted over those 60 s by stethoscope would simply record an average of about 135/min (completely normal). Hence, the rationality of recording a single average figure of FHR over 60 s seems highly questionable and probably unsafe when a hand held Doppler device gives a contemporaneous FHR and its trend over the time of auscultation. The World Health Organization (WHO) guidelines simply recommend counting FHR over 1 min (not related to contraction) with a view to diagnosing persistent bradycardia (below 120) or tachycardia (over 160), but do not make any claim at all to detect late decelerations [13]. The WHO also makes it clear that its guidelines are practical and relevant mainly to the birth attendants with limited skill-sets in the rural health centres in the developing countries where hand held Doppler FHR recorders are not available (or often non-functioning). The birth attendants with higher skill-sets in the resource rich developed countries can of course do better.

## Improve IA or Switch Over to CTG?

The author has come across a common body of (expert) opinion that rather than debating or improving the reliability of IA, it should be converted to continuous CTG after 1 h of pushing in the second stage (e.g. in the cases described above) as a better method of fetal monitoring and to assist transfer decisions and intervention. However, switching over to CTG is often not possible in the community settings even in developed countries. The flawed rationale behind this seems that a poor technique of fetal monitoring is somehow acceptable in the first stage and first hour of the second stage of labor. Moreover, switching over to CTG after 1 h of second stage is categorically discouraged by NICE for fear of increasing unnecessary operative intervention, unless there is an additional complication [1]. The arbitrary watershed of “1 h” for prolonged second stage of labor is also debatable [1]. Fetal hypoxia in many cases starts in the late first stage of labor, hence should its detection await a better technique of CTG to be started after 1 h of pushing in the second stage? Thus any potential to improve IA should be subjected to a wide debate. The option of switching over to CTG should not be an excuse to accept or ignore major correctible flaws of IA.

## Review of Relevant Literature and Rationale for Improvement

Currently the IA can pick up some pathological FHR decelerations but this may be due to fortuitous more extended auscultation. Some senior midwives in the UK state that they perform more prolonged FHR auscultation in addition to the recommended 60 s by the current guidelines anyway, but this is not a common practice. Moreover, this would be an argument in favor of formally extending, clarifying and correcting the current guidelines of IA. Some would argue that the narrative presented above is simply anecdotal and needs to be supported by good quality scientific studies on validity of IA. However, the timing and frequency of auscultation in guidelines for IA are almost entirely a theoretical logical construct (based on level IV evidence) [8, 12]. “Acceptable evidence” in some instances could simply be rational analysis and sound observation from clinical practice, especially when RCTs are hard to conduct in a field like IA. An ideal or perfect option would be to undertake an RCT comparing the two different methods of IA. Not only this would need a very large number of subjects and multiple centers, a particular method will have to be assigned to a particular center to avoid the effect of “learnt behavior” (unintentionally applying elements of a perceived better method). Such RCTs are also very expensive and may give inconclusive results. It is unlikely that such a study or RCT will be conducted. The literature search shows that there are almost no studies/trials performed on IA during labor (excluding addition of “admission CTG”) in the last 15 years, although there are a handful of commentaries, guidelines and analyses. The ubiquitous need for more research should not preclude the lessons from obvious observable fallacies or from logical analysis. Such an exclusion would represent misapplication of evidence-based medicine. A further counter-argument could be that IA has already been shown to be as good as continuous CTG in the meta-analysis of RCTs [7]. But this may apply to more extended IA because the constituent RCTs by Luthy et al (1987) and Vintzileos et al (1993) included more extended timing of FHR auscultation, i.e. during or in-between and after contractions every 30 min [14, 15]. Devane et al (2012) published systematic review of trials of IA with or without “admission CTG” [16]. This review showed no benefit with addition of “admission CTG”, but interestingly some of the constituent trials used IA for a minimum of one minute “during and immediately following” a contraction [17]. The timing of IA before (or during) and after contractions seems more crucial than the frequency of auscultation. If a more appropriately prolonged IA is performed before and after 2 - 3 contractions every time, then it seems difficult to justify why IA should be performed every 15 min in the first stage and every 5 min in the second stage, a very onerous demand indeed [12]. It is also not patient-friendly and seems to place undue excessive burden on birth attendants with unfair medico-legal repercussions [12].

## Conclusions

IA of FHR is an accepted and often favored mode of fetal monitoring in low risk labors [1, 8-10]. The use of IA is likely to increase in future and could be suitable for almost 50% of labors [1]. All national guidelines for regimes of IA are based on the expert consensus only and one of the foremost aims is to detect late decelerations [1]. The two anonymized cases (out of many encountered) with birth asphyxia presented suggest that auscultating for 60 s after contractions may commonly comprise the recovery phase of already established late or variable decelerations when present. This may be mistaken for normal or accelerative FHR pattern in the clinical practice. Even a more serious combination of baseline tachycardia and late decelerations could be missed. A recent recommendation of recording a single average figure of FHR over 60 s after contractions [1] seems even less informative and probably unsafe. Hence the improved recommendations for IA should include auscultating for 30 - 60 s before and after contractions (or contraction to contraction) over 2 - 3 contractions in order to ascertain the baseline FHR as well as any decelerations with late timing or recovery. Such an extended auscultation with hand held Doppler device and interpretation of the trend of FHR before and after the contraction is well within the skill-set of trained midwives in developed countries. Auscultation during contraction itself is not essential and has a potential to cause anxiety by detecting clinically insignificant decelerations, but this need not be inevitable as these can be disregarded. FHR decelerations “limited to contraction phase only” (if detected) should be disregarded as these are mostly of a benign reflex nature [5, 6]. Secondly, it should be safely possible to extend the interval between IA to 30 min in the first stage and 10 min in second stage which would be more practical and user/patient-friendly [12]. The FHR just before contractions should be taken as the “baseline FHR”. If the FHR after contractions is more than 15 bpm below this baseline, then more extended auscultation with Doppler should be performed over a few contractions to judge whether there are decelerations with late onset and late recovery. If these are suspected or cannot be ruled out, then a CTG should be commenced. The current methodology of IA as proposed by all national guidelines [1, 8-10] is likely to pose a risk to patients by missing many or most late decelerations. It seems unjustifiable to knowingly accept these relatively easily remediable flaws of IA. In contrast, the model of IA proposed in this paper is more likely to detect majority of pathological (late) decelerations without converting excessive number of IA to EFM, and thus has a potential to enhance the patient care and safety.
